# Ecotoxicological impacts of benzo[a]pyrene on *Ctenopharyngodon idella* larvae: hemato-immunological disruption, genotoxicity, and histopathological alterations

**DOI:** 10.3389/fimmu.2026.1872548

**Published:** 2026-07-07

**Authors:** Mohamed Hamed, Naglaa AbdelAll, Shaimaa K. A. Idriss, Jiezhang Mo, Ghada A. Khouqeer, Christopher J. Martyniuk

**Affiliations:** 1 Department of Zoology, Faculty of Science, Al-Azhar University (Assiut Branch), Assiut, Egypt; 2 Physics Department, Faculty of Science, Imam Mohammad Ibn Saud Islamic University (IMSIU), Riyadh, Saudi Arabia; 3 Department of Aquatic Animal Medicine and Management, Faculty of Veterinary Medicine, Assiut University, Assiut, Egypt; 4 Department of Biology and Guangdong Provincial Key Laboratory of Marine Disaster Prediction and Prevention, Shantou University, Shantou, China; 5 Center for Environmental and Human Toxicology, Department of Physiological Sciences, College of Veterinary Medicine, University of Florida, Gainesville, FL, United States

**Keywords:** benzo[a]pyrene, *Ctenopharyngodon idella*, cytotoxicity, genotoxicity, hematotoxicity, melanomacrophage centers

## Abstract

**Introduction:**

Environmental pollutants are increasingly recognized as critical modulators of fish immune-associated alterations, with significant implications for aquaculture sustainability. Benzo[a]pyrene (B[a]P), a persistent polycyclic aromatic hydrocarbon, represents a major aquatic contaminant capable of inducing immunotoxic effects in early life stages of fish.

**Methods:**

Larvae of grass carp (*Ctenopharyngodon idella*) were exposed to 1, 10, and 100 µmol/L B[a]P for 14 days to investigate the impact of B[a]P on hemato-immunological alterations, cytotoxicity, genotoxicity, and histopathological responses in immune-related organs.

**Results:**

B[a]P induced hematological and cellular alterations associated with possible immunotoxic effects in grass carp larvae. Hematological disruption was accompanied by increased erythrocyte apoptosis, reflecting cytotoxic stress. Genotoxic damage was evident through elevated DNA strand breaks, micronuclei formation, and nuclear abnormalities, suggesting compromised cellular integrity. Histopathological analysis revealed severe alterations in the liver and spleen, including lymphoid depletion, vascular congestion, and pronounced melanomacrophage center (MMC) activation with hemosiderin accumulation, highlighting immune-associated alterations.

**Conclusion:**

Collectively, these findings demonstrate that B[a]P acts as a potent environmental immunotoxicant that disrupts immune-associated biomarkers, induces cytogenotoxic damage, and impairs key immune organs in fish larvae. This study provides insights into how environmental contaminants modulate fish possible immunotoxic effects and underscores the importance of pollution management strategies to support disease resistance and sustainable aquaculture practices.

## 1 Introduction

Polycyclic aromatic hydrocarbons (PAHs) are toxic and persistent organic pollutants produced by incomplete combustion of organic matter. Benzo[a]pyrene (B[a]P), a five-ring PAH, is a priority environmental contaminant due to its carcinogenic, mutagenic, and teratogenic effects (1, 2). Its highly lipophilic structure (log Kow = 6.04) promotes bioaccumulation and interaction with biological membranes, posing substantial risks to aquatic organisms, particularly fish (3).

B[a]P is widely distributed in air, soil, water, and sediments due to its persistence and long-range transport. It is mainly released from anthropogenic sources associated with incomplete combustion, including vehicle exhausts, industrial emissions, and coal- or oil-fired power plants, with coal combustion being a major contributor (1, 4–6). In aquatic environments, B[a]P contamination arises from atmospheric deposition, industrial discharge, oil spills, and urban runoff, with concentrations in surface water typically ranging from 0.1 to 100 ng/L, and up to 8.61 ng/L in some areas (7–9). Sediment concentrations can be significantly higher, reaching up to 5,000 ng/g in polluted regions like China’s Bohai Sea (10, 11). In heavily industrialized zones, surface waters have recorded B[a]P levels between 200–2512 ng/L, with sediments often exceeding 5732 ng/g dry weight (12–14). Conversely, pristine freshwater bodies usually contain B[a]P levels from 100 to 219 ng/L, primarily from atmospheric sources (15, 16). Drinking water standards in the EU set a B[a]P limit of 10 ng/L, though some samples have reported up to 36.3 ng/L (17). EPA guidelines recommend a maximum of 0.2 ng/L for aquatic life protection (18), a threshold often exceeded in rivers receiving untreated industrial waste.

Exposure to B[a]P induces severe toxic effects in fish across molecular, cellular, physiological, and population levels. At the molecular level, B[a]P metabolism generates reactive oxygen species (ROS), leading to oxidative stress, lipid peroxidation, and DNA damage (19–21). These effects trigger apoptosis, mitochondrial dysfunction, immune-associated alterations, and hematological disturbances such as anemia and leukopenia (22–24). These effects trigger apoptosis, mitochondrial dysfunction, responses associated with potential immunotoxicity, and hematological disturbances such as anemia and leukopenia (25–27). B[a]P also exerts cytotoxic and genotoxic effects, including DNA strand breaks, micronuclei formation, and nuclear abnormalities (28–31).

Toxicity is largely mediated through activation of the aryl hydrocarbon receptor (AhR) pathway and induction of CYP1A enzymes, which metabolize B[a]P into reactive intermediates such as BPDE that form DNA adducts and mutations (29, 32). At higher biological levels, B[a]P exposure causes developmental defects, behavioral alterations, reduced fecundity, and increased mortality, ultimately threatening fish population stability (33–35). Concurrently, B[a]P metabolism produces excess ROS, causing oxidative stress, apoptosis, and hematological disorders, ultimately leading to cellular dysfunction, tissue damage, and impaired physiological performance (25, 29, 33).

Immune-associated biomarkers represent the primary line of defense in fish, providing rapid, non-specific protection against invading pathogens and environmental stressors (36, 37). It involves a coordinated network of cellular and humoral components, including leukocytes, phagocytic activity, and immune-related organs such as the spleen and liver (38, 39). Importantly, innate immune responses are highly sensitive to environmental conditions, making them key indicators of pollutant-induced immunotoxicity in aquatic organisms.

To assess B[a]P toxicity in grass carp larvae, this study used a range of biomarkers encompassing physiological, cytotoxic, genotoxic, and histopathological endpoints. Hematological parameters were measured to evaluate systemic toxicity and immune function, serving as sensitive indicators of PAH-induced stress (40–42). Cytotoxicity was assessed via apoptosis assays (43), while genotoxicity was evaluated using the comet assay, micronucleus test, and nuclear abnormality analysis in red blood cells, providing sensitive measures of DNA and chromosomal damage (44, 45). Histopathological changes were also examined, serving as robust indicators of pollutant impact and overall population health in the ecosystem (46, 47).

Grass carp (*Ctenopharyngodon idella*), an herbivorous freshwater fish, is important in aquaculture and ecosystem management (48). Its rapid growth, widespread distribution, and economic value make it an ideal sentinel for aquatic toxicology (49, 50). Larvae are especially vulnerable due to underdeveloped detoxification systems, permeable integuments, and high metabolic demands, making them sensitive indicators of environmental contaminants (51, 52). Their manageable size and well-characterized development also make them suitable for laboratory exposures, supporting studies of B[a]P toxicity for aquaculture, ecological risk assessment, and conservation (52, 53).

The primary objective of this study was to elucidate the sublethal effects of B[a]P exposure on the physiological, cytotoxic, and genotoxic responses of grass carp larvae. Specifically, the study aimed to (i) characterize alterations in hematological profiles, (ii) quantify erythrocyte apoptosis as an indicator of cytotoxicity, and (iii) assess DNA damage and chromosomal aberrations as markers of genotoxicity. The central hypothesis was: (1) B[a]P nominal exposure would induce hematological and disruptions reflective of physiological stress, (2) B[a]P would trigger apoptosis in erythrocytes, indicating cellular toxicity, and (3) B[a]P would cause significant DNA damage and nuclear abnormalities, demonstrating its genotoxic potential. Through these comprehensive investigations, this study endeavors to advance our understanding of the toxicodynamic effects of B[a]P in early life stages of freshwater fish, with implications for environmental health protection and pollution management strategies.

## 2 Materials and methods

### 2.1 Chemicals

Benzo[a]pyrene (B[a]P; ≥96% purity) was obtained from Sigma-Aldrich (St. Louis, MO, USA). A stock solution of B[a]P was prepared by dissolving the compound in dimethyl sulfoxide (DMSO; analytical grade, Sigma-Aldrich) and stored in the dark at 4 °C to prevent degradation. The final concentration of DMSO in exposure tanks did not exceed 0.01%, a level considered non-toxic to fish (54).

### 2.2 Fish husbandry and acclimation

One-month-old grass carp (*Ctenopharyngodon idella*) larvae (average weight: 5 ± 1 g) were sourced from a local aquaculture farm verified to be disease-free. Prior to the experimental exposure, larvae underwent a 14-day acclimation period under controlled laboratory conditions. Fish were maintained in aerated, dechlorinated freshwater tanks (capacity: 100 L) at a temperature of 25 ± 1 °C, pH 7.2 ± 0.2, with a 12:12 h light-dark photoperiod. Dissolved oxygen levels were maintained above 80% saturation through continuous aeration. During acclimation, fish were fed a commercial pelleted diet (SKRETTING, Egypt) twice daily consisting of 30% crude protein, 6% crude lipid, 10% crude fiber, 12% moisture, and 5% ash, supplemented with vitamins and minerals. Water quality parameters (ammonia, nitrite, nitrate) were monitored daily and maintained within optimal ranges for larval development.

### 2.3 Experimental design

Following acclimation, grass carp larvae were randomly assigned to four experimental groups a control group (water with DMSO concentration was kept at 0.01%) and three treatment groups exposed to B[a]P at concentrations of 1, 10, and 100 µmol/L (55–57). Each treatment consisted of three replicate tanks containing six fish per tank (18 fish per treatment). Exposure was therefore applied at the tank level under semi-static conditions.

Because B[a]P is highly hydrophobic, adsorption to tank surfaces, suspended particles, and organic matter may have influenced dissolved exposure concentrations during the experiment. Therefore, all reported concentrations represent nominal concentrations. However, fresh exposure solutions were prepared daily under semi-static conditions with 50% water renewal to maintain relatively stable exposure conditions (46). Fish were exposed to their respective treatments for a period of 14 days under semi-static conditions. During the exposure period, fish were fed twice daily with a commercial diet, and uneaten food and waste were removed promptly to maintain water quality (47). The selected nominal exposure concentrations of B[a]P (1, 10, and 100 µmol/L) were based on previous toxicological studies that have successfully demonstrated physiological, and genotoxic responses in fish models e.g., (55–57). Although dissolved concentrations of B[a]P are generally reported in the ng/L range, higher experimental concentrations are commonly employed in toxicology studies to characterize sublethal hazard responses, concentration-dependent biological effects, and underlying toxicodynamic pathways in sensitive early life stages of aquatic organisms. Therefore, the present exposure regime should primarily be interpreted as a controlled hazard-identification and exposure model rather than a direct simulation of typical environmental water concentrations.

For downstream analyses, fish were randomly sampled from replicate tanks to minimize tank-related sampling bias. Hematological analysis, apoptosis assay, comet assay, erythrocyte abnormality assessment, and histopathological examinations were conducted using six fish per treatment group (two fish randomly sampled from each tank).

### 2.4 Hematological analysis

At the end of the exposure period, larvae were anesthetized using ice, and blood samples were collected from the caudal vein, approximately 200–300 µL of blood was collected from each fish using heparinized microcapillary tubes immediately after euthanasia. Samples were maintained at 4 °C and analyzed within 6 h. Red Blood Cell (RBC) and White Blood Cell (WBC) counts were determined using a hemocytometer under a light microscope (Olympus BX51) (58). Hemoglobin (Hb) concentration was measured using the cyanmethemoglobin method, and Hematocrit (Ht) was assessed via capillary hematocrit tube (59). Mean Corpuscular Volume (MCV), Mean Corpuscular Hemoglobin (MCH), and Mean Corpuscular Hemoglobin Concentration (MCHC) were calculated using standard formulas (60). Differential leukocyte counts (Monocyte %, Neutrophil %, Lymphocyte %) were performed on Giemsa-stained blood smears by counting 200 cells per slide under a microscope.

### 2.5 Apoptosis assay

Eryptosis was detected following the procedures described by Sayed (61). Briefly, blood smears were prepared and stained with acridine orange (AO). Stained erythrocytes were examined using a fluorescence microscope (Zeiss Axioplan2) equipped with a digital 3CCD color video camera (Sony, AVT-Horn, Japan). For each sample, 500 erythrocytes were manually counted under blinded conditions according to standardized fluorescence and morphological criteria. Images were captured to visualize normal (yellow circles) and apoptotic cells (red circles) (61, 62).

### 2.6 Comet assay (DNA damage)

DNA damage in RBCs was evaluated using the single-cell gel electrophoresis (comet assay) under alkaline conditions. RBCs were isolated as described above, mixed with 0.5% low-melting-point agarose, and layered onto slides pre-coated with 1% normal-melting-point agarose. Slides were lysed in a solution (2.5 M NaCl, 100 mM EDTA, 10 mM Tris, 1% Triton X-100, pH 10) for 1 h at 4 °C, followed by unwinding in alkaline buffer (300 mM NaOH, 1 mM EDTA, pH >13) for 20 min. Electrophoresis was performed at 25 V (300 mA) for 20 min in the same buffer. Slides were neutralized with 0.4 M Tris (pH 7.5), stained with 20 µg/mL ethidium bromide, and examined under a Zeiss Axioplan2 fluorescence microscope (200×) with a digital 3 CCD color video camera (Sony, AVT-Horn). The Tail Moment Score (%) was quantified using CASP software by analyzing 100 cells per sample (63–65).

### 2.7 RBC alterations assay and nuclear abnormalities

Blood smears were prepared from caudal vein samples of larvae, air-dried, fixed in methanol for 10 min, and stained with H&E. The RBC alterations assay and nuclear abnormalities including (crenated, acanthocyte, teardrop, elliptocyte, echinocyte, spherocyte, sickle, and micronuclei) were identified and counted by examining 10000 erythrocytes per fish (1000 erythrocytes per slide) under a light microscope (Olympus BX51) at 1000× magnification. Micronuclei were identified according to standard cytogenetic criteria, including rounded morphology, distinct nuclear boundaries, staining characteristics similar to the primary nucleus, absence of refractility, and clear separation from the main nucleus. Structures associated with cell degeneration, nuclear smearing, diffuse staining, or poorly preserved erythrocytes were excluded from analysis to minimize scoring artifacts. Results were expressed as the number of affected cells per 1000 erythrocytes. Representative images were captured to document morphological changes (62, 66).

### 2.8 Histological and histochemical examination

Tissue samples (liver and spleen) were dissected from each fish and rinsed with neutral saline. Six fish were examined per treatment group, with two fish randomly sampled from each replicate tank. Samples were fixed in 10% neutral buffered formalin, dehydrated through a graded alcohol series, cleared with methyl benzoate, and embedded in paraffin wax. Sections of 5 µm thickness were cut and stained using hematoxylin and eosin (H&E) (67). In addition, Prussian blue stain, a diagnostic histochemical method, was applied to identify and evaluate iron deposits in liver and spleen tissues, highlighting iron as a distinct blue coloration. For Prussian blue staining, tissue sections were deparaffinized, rehydrated through graded ethanol series, and incubated with freshly prepared Perl’s Prussian blue solution consisting of equal volumes of 2% potassium ferrocyanide and 2% hydrochloric acid. Sections were subsequently rinsed in distilled water and counterstained with nuclear fast red before dehydration and mounting. All stained sections were examined and photographed using an Olympus CH30 microscope. Histopathological lesions were semi-quantitatively graded as absent (−): no detectable lesion, mild (+): focal or minimal alteration affecting <25% of the observed tissue area, moderate (++): multifocal alteration affecting approximately 25–50% of the tissue area, severe (+++): extensive alteration affecting approximately 50–75% of the tissue area, very severe (++++): diffuse or widespread alteration affecting >75% of the tissue area (**Table 1**) (68).

**Table 1:** Semi-quantification of histopathological lesions in liver and spleen tissues of grass carp larvae (*Ctenopharyngodon idella*) following 14-day exposure to benzo[a]pyrene (B[a]P) at concentrations of 1, 10, or 100 µmol/L.

Lesions	Control	1 µmol/L	10 µmol/L	100 µmol/L
Liver				
Hepatic cord disarray	–	+++	+++	++++
Vacuolar degeneration of hepatocytes	–	++	+++	+++
Fatty degeneration of hepatocytes	–	–	+	++
Necrosis of hepatocytes	–	+	++	+++
separation of epithelial cells from basement membrane in portal area	–	+++	++	+++
Sinusoidal dilatation	–	++	++	+++
Central vein dilatation and congestion	–	++	+++	++++
Congestion of hepatic sinusoids	–	+++	++	++++
Congestion of hepatopancrease	–	++	+++	++++
Vascular congestion with coagulated blood elements	–	+	++	+++
Spleen				
Hyperplasia of lymphoid follicles	–	+++	+++	++
Lymphoid exhaustion of white pulp	–	++	++++	++++
Depletion of lymphocytes	–	++	+++	++++
Dilatation of blood vessels lumen	–	+	++	+++
Congestion of blood vessels	–	+	+	++
Congestion of red pulp	–	+	++	+++
Melanomacrophages aggregation	–	+++	+++	++++
Hemosiderin precipitation	–	++	++	+++

Lesion severity was evaluated microscopically and scored using a semi-quantitative scale based on the extent and intensity of pathological alterations. Histopathological lesions were semi-quantitatively graded as absent (−): no detectable lesion, mild (+): focal or minimal alteration affecting <25% of the observed tissue area, moderate (++): multifocal alteration affecting approximately 25–50% of the tissue area, severe (+++): extensive alteration affecting approximately 50–75% of the tissue area, very severe (++++): diffuse or widespread alteration affecting >75% of the tissue area.

### 2.9 Statistical analysis

All data were expressed as mean ± standard deviation (SD). Statistical analyses were performed using GraphPad Prism version 9.0 (GraphPad Software, San Diego, CA, USA). Normality and homogeneity of variances were assessed using the Shapiro-Wilk test. Individual fish values were used as biological replicates for statistical analyses. Continuous parametric data, including hematological parameters, apoptosis assay results, comet assay data, and erythrocyte abnormality frequencies, were analyzed using one-way analysis of variance (ANOVA) followed by Tukey’s multiple-comparison *post hoc* test. The significance level of p < 0.05 was considered statistically significant. Because exposure was applied at the tank level, potential tank effects cannot be completely excluded; however, replicate tanks were maintained under identical experimental conditions to minimize tank-to-tank variability.

## 3 Results

### 3.1 Hematological parameters

Exposure to B[a]P at concentrations of 1, 10, and 100 µmol/L significantly altered hematological parameters in grass carp (*Ctenopharyngodon idella*) (**Figure 1**). RBC count declined markedly across all exposure groups (one-way ANOVA, F_3,20_ = 21.39, *p* < 0.0001), with *post hoc* analysis showing marked declines at all exposure concentrations compared with the control (p < 0.0001). Similarly, significant reductions were observed in Hb concentration (F_3,20_ = 9.13, *p* = 0.0005) and Ht (F_3,20_ = 10.96, *p* = 0.0002), with significant decreases observed at 1 (p < 0.001), 10 (p < 0.0001), and 100 µmol/L (p < 0.0001). MCV increased significantly (F_3,20_ = 4.62, *p* = 0.0129), whereas MCH (F_3,20_ = 1.02, *p* = 0.38) and MCHC (F_3,20_ = 0.50, *p* = 0.55) were not significantly affected. Total WBC counts were significantly reduced (F_3,20_ = 5.50, *p* = 0.007), with *post hoc* analysis indicating significant decreases at 100 µmol/L (p < 0.0001), accompanied by significant alterations in leukocyte differentials. Lymphocyte percentages increased significantly (F_3,20_ = 11.54, *p* = 0.0001), with significant elevations at 10 (p < 0.01) and 100 µmol/L (p < 0.0001), while monocyte percentages declined (F_3,20_ = 4.66, *p* = 0.012). In contrast, neutrophil percentages were not significantly affected by B[a]P exposure (F_3,20_ = 0.32, *p* = 0.80).

**Figure 1:**
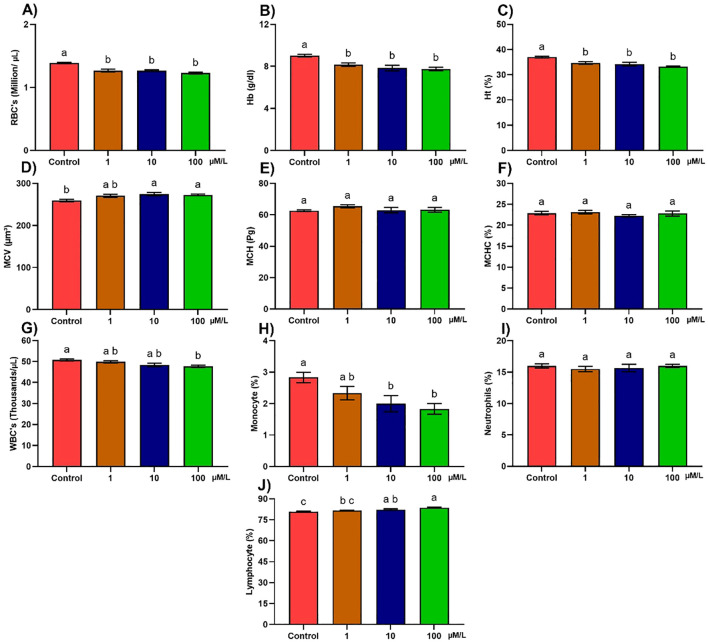
Effects of Benzo[a]pyrene (B[a]P) exposure on hematological parameters of grass carp (*Ctenopharyngodon idella*) larvae after 14 days at concentrations of 1, 10, or 100 µmol/L. The evaluated parameters include: **(A)** Red Blood Cell count (RBC; million/µL), **(B)** Hemoglobin concentration (Hb; g/dL), **(C)** Hematocrit (Ht; %), **(D)** Mean Corpuscular Volume (MCV; fL), **(E)** Mean Corpuscular Hemoglobin (MCH; pg), **(F)** Mean Corpuscular Hemoglobin Concentration (MCHC; %), **(G)** White Blood Cell count (WBC; thousands/µL), **(H)** Monocyte percentage (%), and **(I)** Neutrophil percentage (%). Data are presented as mean ± standard deviation (SD). Statistical differences were assessed using one-way ANOVA followed by Tukey’s multiple-comparison test. The letters indicate significant differences from the control group.

### 3.2 Apoptotic cell response

B[a]P exposure induced significant apoptosis in grass carp RBC, as assessed by fluorescence microscopy (**Figure 2**). The percentage of apoptotic cells increased markedly among treatments (F_3,20_ = 270.3, *p* < 0.0001). *Post hoc* analysis revealed significant elevations in apoptotic cell percentages at 1 µmol/L (*p* < 0.0001) and at 10 and 100 µmol/L (*p* < 0.0001) compared with the control group (**Figure 2A**). In the control group (**Figure 2B**), fluorescence images showed predominantly normal cells (yellow circles), while at 1 µmol/L (**Figure 2C**), the number of apoptotic cells (red circles) increased. This trend intensified at 10 µmol/L (**Figure 2D**), with a further increase in apoptotic cells, and peaked at 100 µmol/L (**Figure 2E**), where the highest number of apoptotic cells was observed, indicating severe cellular toxicity and programmed cell death triggered by B[a]P.

**Figure 2:**
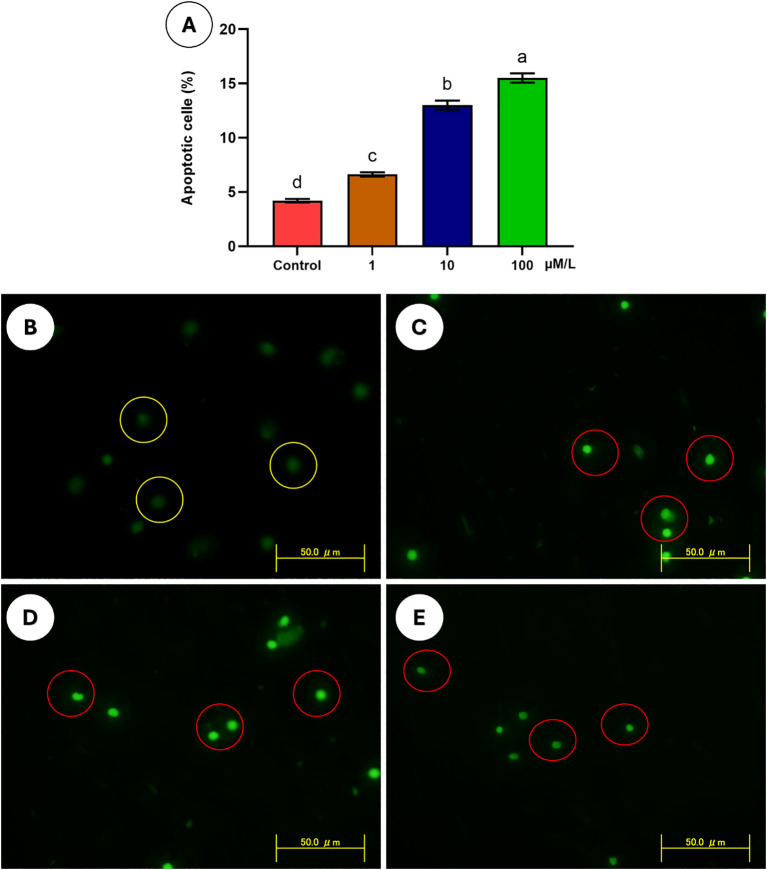
Apoptotic cell response in red blood cells of grass carp (*Ctenopharyngodon idella*) following 14-day exposure to benzo[a]pyrene (B[a]P) at concentrations of 1, 10, or 100 µmol/L. **(A)** shows the percentage of apoptotic cells (%), while **(B–E)** depict fluorescence microscopy images of red blood cells: **(B)** control group with normal cells (indicated by yellow circles), and **(C–E)** increasing levels of apoptotic cells (red circles) observed at B[a]P concentrations of 1, 10, and 100 µmol/L, respectively. Data are presented as mean ± standard deviation (SD). Statistical differences were assessed using one-way ANOVA followed by Tukey’s multiple-comparison test. The letters indicate significant differences from the control group.

### 3.3 DNA damage (comet assay)

The comet assay revealed significant DNA damage in grass carp red blood cells following B[a]P exposure (**Figure 3**). The tail moment score, an index of DNA strand breaks, was significantly affected by B[a]P exposure (F_3,20_ = 11.85, *p* = 0.0001). *Post hoc* analysis revealed significant increases in tail moment score at 10 µmol/L (*p* < 0.001), and 100 µmol/L (*p* < 0.0001) compared with the control group, demonstrating a concentration-related toxicological responses under nominal exposure conditions indicated pronounced genotoxic responses (**Figure 3A**). Fluorescence images showed minimal comet tails in the control group (**Figure 3B**), indicating intact DNA. At 1 µmol/L (**Figure 3C**), comet tails lengthened slightly, while at 10 µmol/L (**Figure 3D**), the tails became more pronounced, and at 100 µmol/L (**Figure 3E**), the longest comet tails were observed, confirming the highest level of DNA damage and B[a]P’s genotoxic potential.

**Figure 3:**
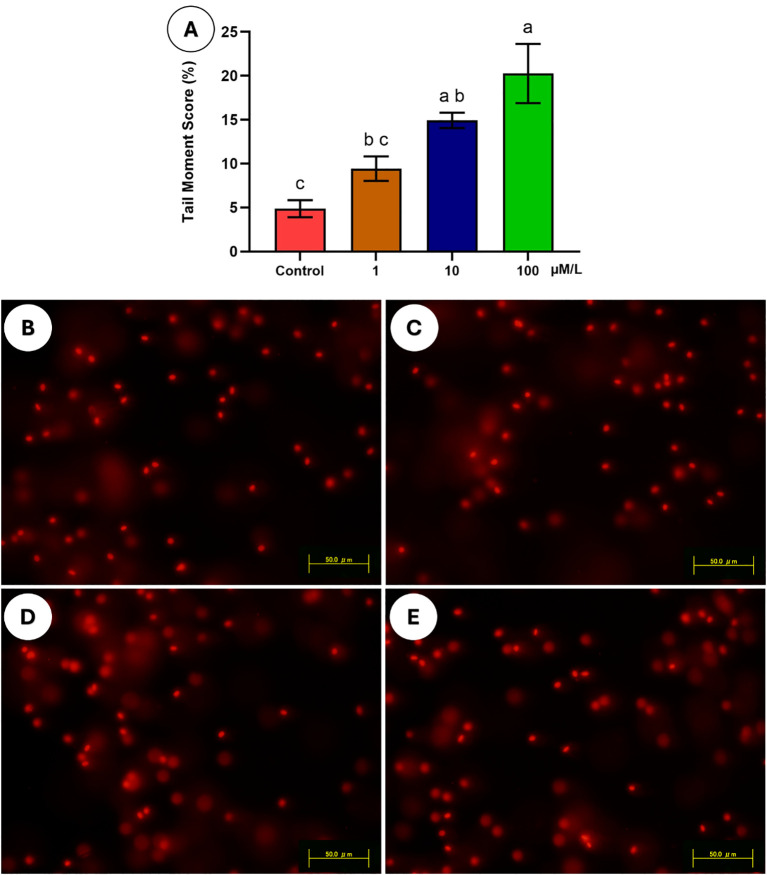
DNA damage assessment in red blood cells of grass carp (*Ctenopharyngodon idella*) using the comet assay following 14-day exposure to benzo[a]pyrene (B[a]P) at concentrations of 1, 10, or 100 µmol/L. **(A)** displays the Tail Moment Score (%), a quantitative indicator of DNA strand breaks, while **(B–E)** show representative comet assay fluorescence images: **(B)** control group with minimal DNA migration and **(C–E)** increasing comet tail lengths at 1, 10, or 100 µmol/L B[a]P, respectively, indicating progressive DNA damage. Data are presented as mean ± standard deviation (SD). Statistical differences were assessed using one-way ANOVA followed by Tukey’s multiple-comparison test. The letters indicate significant differences from the control group.

### 3.4 RBC alterations assay and nuclear abnormalities

B[a]P exposure caused significant genotoxic effects in grass carp erythrocytes, as evaluated by the micronucleus assay (**Figure 4**). The frequency of RBC alterations increased significantly among treatments (F_3,20_ = 1214, *p* < 0.0001), and nuclear abnormalities were also significantly elevated (F_3,20_ = 145, *p* < 0.0001). *Post hoc* analysis showed significant increases in RBC alterations at 1 µmol/L (*p* < 0.001), at 10 and 100 µmol/L (*p* < 0.0001) compared with the control group (**Figure 4A**). Similarly, nuclear abnormalities increased significantly at 10 and 100 µmol/L (*p* < 0.0001) (**Figure 4B**), indicating B[a]P-induced chromosomal damage. Microscopy images of the control group showed normal red blood cells (RBCs) (**Figure 4C**), while at 1 µmol/L (**Figure 4D**), abnormalities such as crenated, acanthocyte, teardrop, elliptocyte, and echinocyte cells appeared. At 10 µmol/L (**Figure 4E**), these abnormalities increased, and at 100 µmol/L (**Figure 4F**), the highest frequency of abnormalities, including spherocytes, sickle cells, and micronuclei, was observed, confirming B[a]P ‘s genotoxic impact.

**Figure 4:**
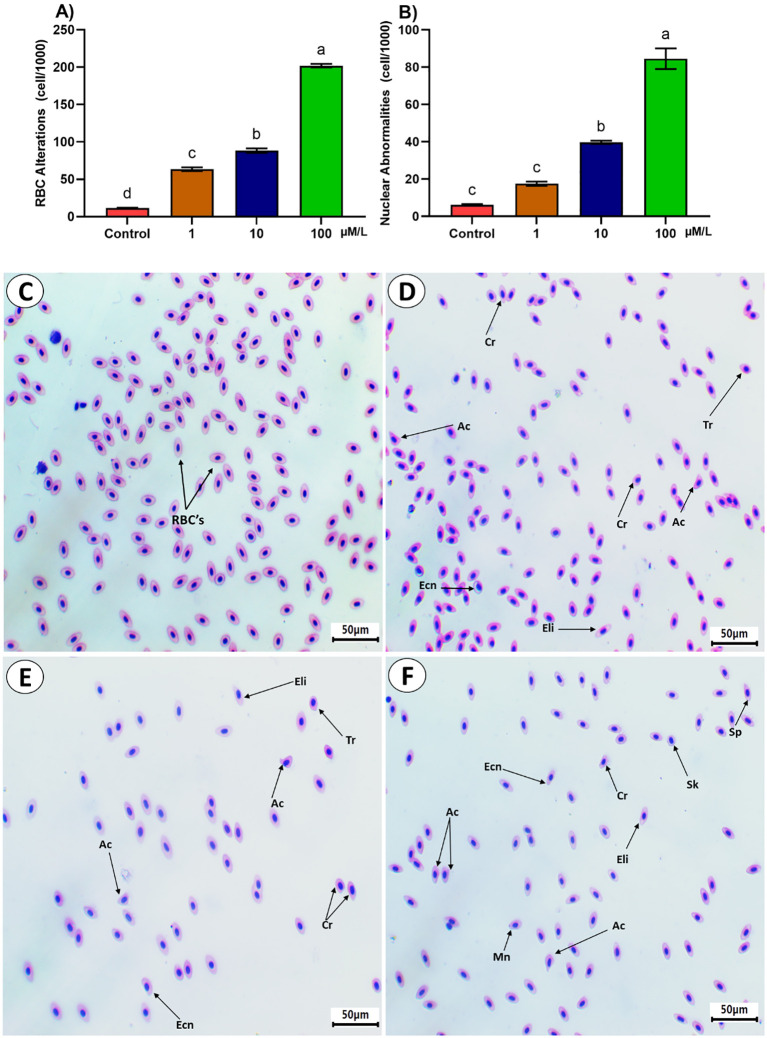
Genotoxic effects of benzo[a]pyrene (B[a]P) on erythrocytes of grass carp (*Ctenopharyngodon idella*) following 14-day exposure to concentrations of 1, 10, or 100 µmol/L. **(A)** frequency of red blood cell (RBC) morphological alterations, and **(B)** incidence of nuclear abnormalities. Panels **(C–F)** display representative erythrocyte images from the control group **(C)** and B[a]P-treated groups at 1 **(D)**, 10 **(E)**, and 100 µmol/L **(F)**, respectively. Observed abnormalities include crenated cells (Cr), acanthocytes (Ac), teardrop cells (Tr), elliptocytes (ELI), echinocytes (ECN), spherocytes (Sp), sickle cells (Sk), and micronuclei (MN). Data are presented as mean ± standard deviation (SD). Statistical differences were assessed using one-way ANOVA followed by Tukey’s multiple-comparison test. The letters indicate significant differences from the control group.

### 3.5 Histological and histochemical of liver

Normal histological structure of liver sections was observed in the control group in comparison with treated groups (**Figure 5A**; **Table 1**). Our histopathological analysis on liver tissues of grass carp exposed to three doses of B[a]P revealed significant loss of normal architecture of hepatocytes in all groups in comparison with control group (**Figure 5B**). After exposure to all doses, vacuolar degeneration of hepatocytes was observed. After exposure to 100 µmol/L hepatic vacuolar degeneration noticed in a severe degree accompanied with multifocal area of fatty degeneration (**Figure 5C**) but in 1 and 10 µmol/L group noticed in a mild degree. Hepatic sinusoidal congestion was observed in a mild degree in all exposed groups (**Figure 5D**). There were a considerable dilatation and congestion in blood vessels and sinusoids were noticed obviously in high degree after exposure to 10 and 100 µmol/L group but decreased to moderate degree in 1 µmol/L group **Figure 5E**. In addition, multifocal areas of hepatic necrosis and apoptosis were cleared with increased exposure in the matching degree as congestion (**Figures 5F, G**). Vascular inflammatory changes in portal area were detected in a severe manner in 100 µmol/L group. Furthermore, degeneration and separation of epithelial cells from basement membrane of blood vessels wall were detected also, vascular congestion with coagulated blood elements was observed clearly in central vein and hepatocyte after exposure to high dose but rarely after exposure to 1 µmol/L group figure (**Figure 5H**).

**Figure 5:**
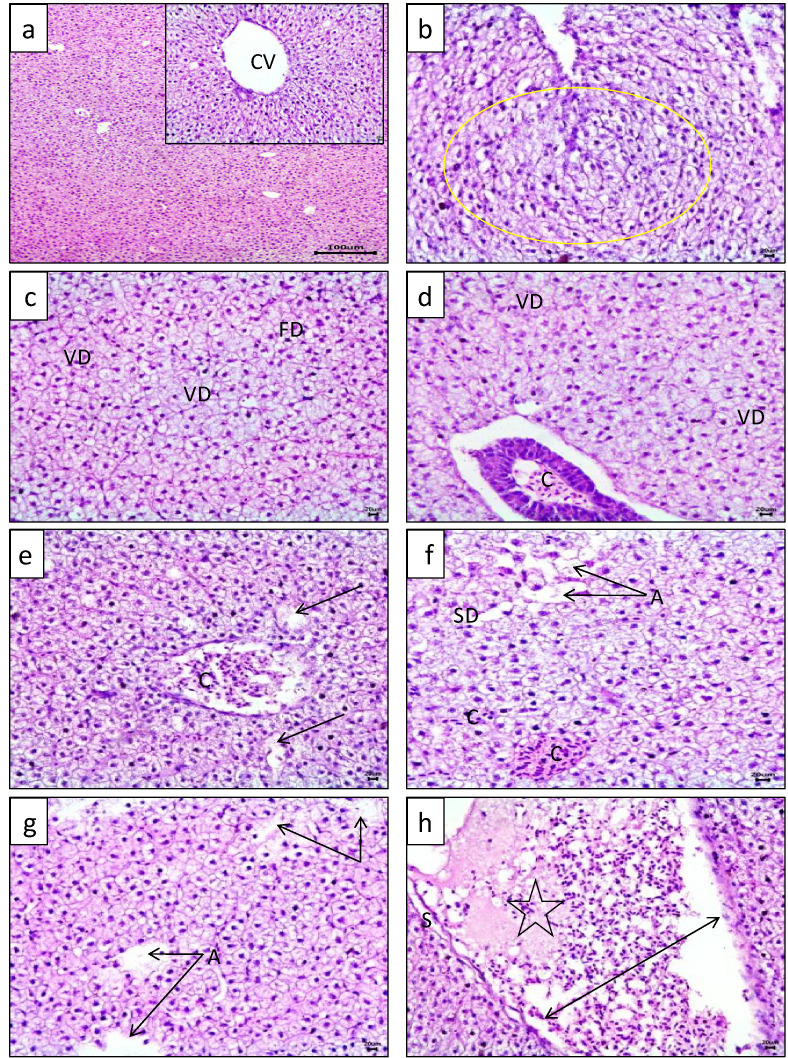
Photomicrographs of liver tissue in grass carp (*Ctenopharyngodon idella*) following 14-day exposure to benzo[a]pyrene (B[a]P) at concentrations of 1, 10, or 100 µmol/L. **(A)**, Control group showing normal hepatic architecture with intact hepatocytes and central vein (CV). **(B)**, B[a]P-exposed groups showing loss of normal hepatocyte architecture. **(C, D)**, Vacuolar degeneration (VD) and fatty degeneration (FD) of hepatocytes with mild to severe hepatocyte congestion **(C)**, particularly evident at 100 µmol/L. **(E)**, Multifocal hepatic necrosis (arrow) and apoptosis **(A)**, along with central vein congestion **(C)**. **(F, G)**, Marked dilation and congestion **(C)** of central veins and sinusoids (SD), accompanied by necrosis and apoptosis **(A)**. **(H)**, Severe vascular alterations in the 100 µmol/L group including vascular congestion with coagulated blood elements (star), extensive dilation (double-headed arrow), and separation (S) of endothelial cells from the basement membrane. Sections were stained with hematoxylin and eosin (H&E); scale bars = 20 µm and 100 µm.

In liver sections, iron is stored in two forms: a soluble form (ferritin) and an insoluble form (hemosiderin). With routine H&E staining, hemosiderin appears as dark brown refractile granules, whereas ferritin is not visible. In contrast, Prussian blue staining highlights hemosiderin as coarse blue granules and ferritin as a faint blue cytoplasmic coloration within hepatocytes (**Figure 6**). The Prussian blue reaction was demonstrated in fish from all exposed groups and represented one of the most diagnostic features, particularly in the 100 µmol/L group, which showed pronounced staining as evidence of severe iron overload.

**Figure 6:**
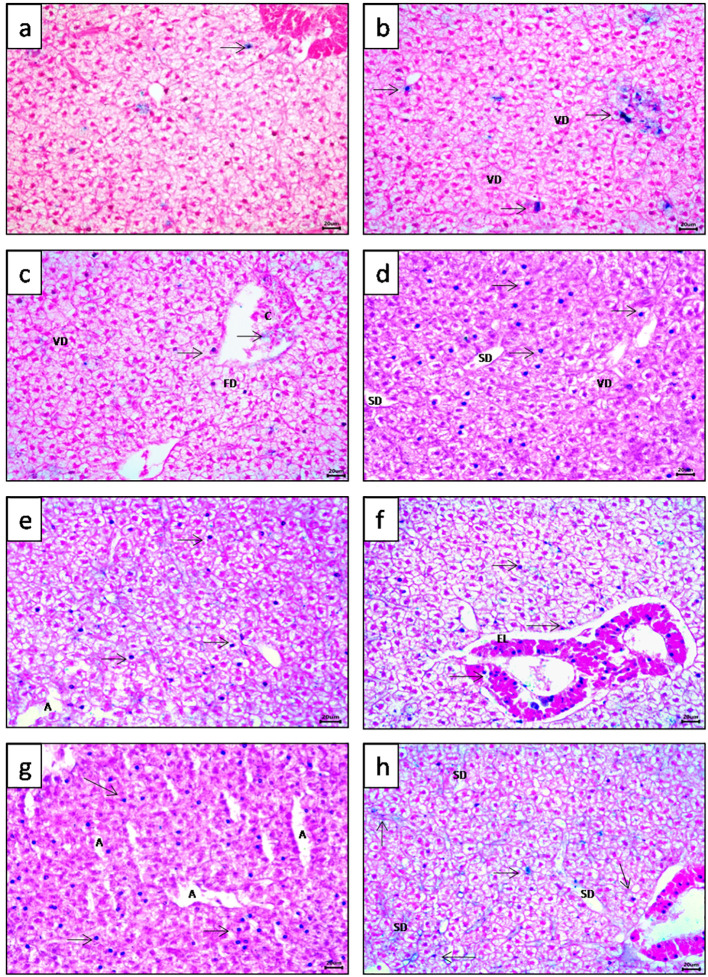
Photomicrographs of Prussian blue–stained liver sections of grass carp larvae (*Ctenopharyngodon idella*) exposed to benzo[a]pyrene (B[a]P) at concentrations of 1, 10, or 100 µmol/L. **(A)** Control group showing absence of iron deposition. **(B, C)** Mild iron accumulation appearing as numerous foci of blue deposits after treatment with 1 µmol/L. **(D, E)** Moderate degree of iron deposition after treatment with 10 µmol/L. **(F–H)** Severe iron overload after treatment with 100 µmol/L. Blue-stained deposits (arrows) indicate sites of iron accumulation. Histopathological changes observed include sinusoidal dilatation (SD), apoptotic areas **(A)**, epithelial lifting (EL), congestion **(C)**, vacuolar degeneration (VD), and fatty degeneration (FD).

### 3.6 Histological and histochemical of spleen

Normal histological structure of spleen sections of grass carp larvae was detected in the control group in contrast with other treated groups (**Figure 7**; **Table 1**). in all treated groups cleared histopathological lesions in spleen were discovered. In 1 µmol/L group, there was mild lymphoid exhaustion of white pulp with moderate hyperplasia of lymphoid follicles, otherwise it became observable to a moderate degree in fish from the 10 µmol/L group (**Figure 7A**). In contrast, multi focal lymphoid follicles hyperplasia were noticed to a moderate degree in fish exposed to 10 µmol/L and to a mild degree in fish from the 100 µmol/L group (**Figures 7B, C**). In fish exposed to 10 or 100 µmol/L, multifocal areas of severe exhaustion and necrosis of lymphocytes were observed clearly with excessive congestion of red pulp in separate areas had also appeared (**Figures 7D, E**). Dilatation and congestion of spleen blood vessels, also noticed to a moderate degree in fish exposed to 10 or 100 µmol/L declined to a mild degree in fish from the 1 µmol/L group (**Figure 7F**). Alternatively, multi focal area of lymphocytic depletion were noticed in a mild degree in fish from the 1 µmol/L group and become apparent in fish from the 100 µmol/L group. On the other hand, in these three groups large focal melanomacrophages (MMC), aggregation was obvious with several serious hemosiderin pigment precipitations in a severe manner, and it was the most diagnostic features noted, especially in fish from the 100 µmol/L group (**Figures 7G, H**).

**Figure 7:**
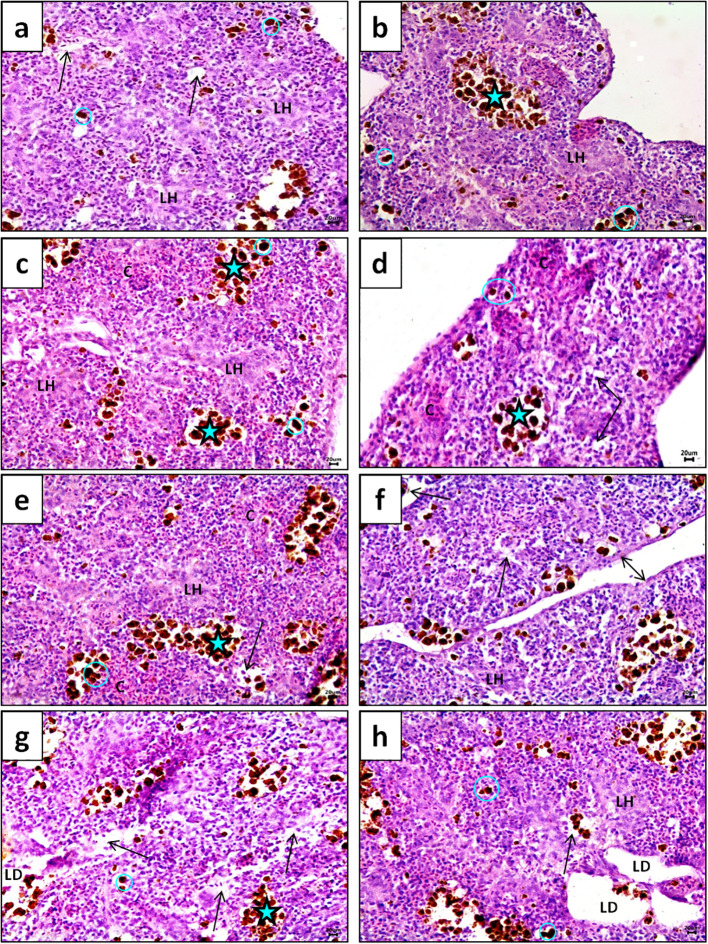
Photomicrographs of spleen tissue in grass carp larvae (*Ctenopharyngodon idella*) following 14-day exposure to benzo[a]pyrene (B[a]P) at concentrations of 1, 10, or 100 µmol/L, showing varying degrees of pathological alterations compared to the control group. **(A)**, focal lymphoid exhaustion in the white pulp (arrow), hyperplasia of lymphoid follicles (LH), and mild hemosiderin pigment deposition (blue circles) were observed in 1µmol/L group. **(B, C)**, Red pulp congestion **(C)**, multifocal hyperplasia of lymphoid follicles (LH), and increased melanomacrophage aggregation (blue stars) with prominent hemosiderin deposits (blue circles) became evident in 100 µmol/L group. **(D, E)**, Extensive congestion of red pulp **(C)**, lymphoid exhaustion (arrow), melanomacrophage accumulation (blue star), and hemosiderin precipitation were noted. **(F)**, Severe dilatation of spleen blood vessels (double head arrow) with lymphoid exhaustion of white pulp (Arrow) and hyperplasia of lymphoid follicle (LH) were clear in third group. **(G)**, In 10 µmol/L group, lymphoid exhaustion of white pulp (Arrow), melanomacrophages aggregation (Blue star) with hemosiderin pigment precipitation (Blue circle) and moderate depletion of lymphocytes (LD) were observable. **(H)**, Multi focal area of lymphocytic depletion (LD), hyperplasia of lymphoid follicle (LH), focal melanomacrophages aggregation include hemosiderin pigment precipitation (Blue circle) and lymphoid exhaustion of white pulp (Arrow), all were detected in 100 µmol/L group. Sections were stained with hematoxylin and eosin (H&E); scale bars = 20 µm µm.

In spleen sections, the brown pigment observed in MMC with H&E stain became dark blue with Prussian blue, confirming the presence of stainable iron (hemosiderin). This finding reflects the breakdown of red blood cells and subsequent iron deposition (**Figure 8**). The Prussian blue reaction was demonstrated in all exposed groups and represented one of the most diagnostic features, particularly in the 100 µmol/L group, which showed pronounced staining as evidence of severe iron overload.

**Figure 8:**
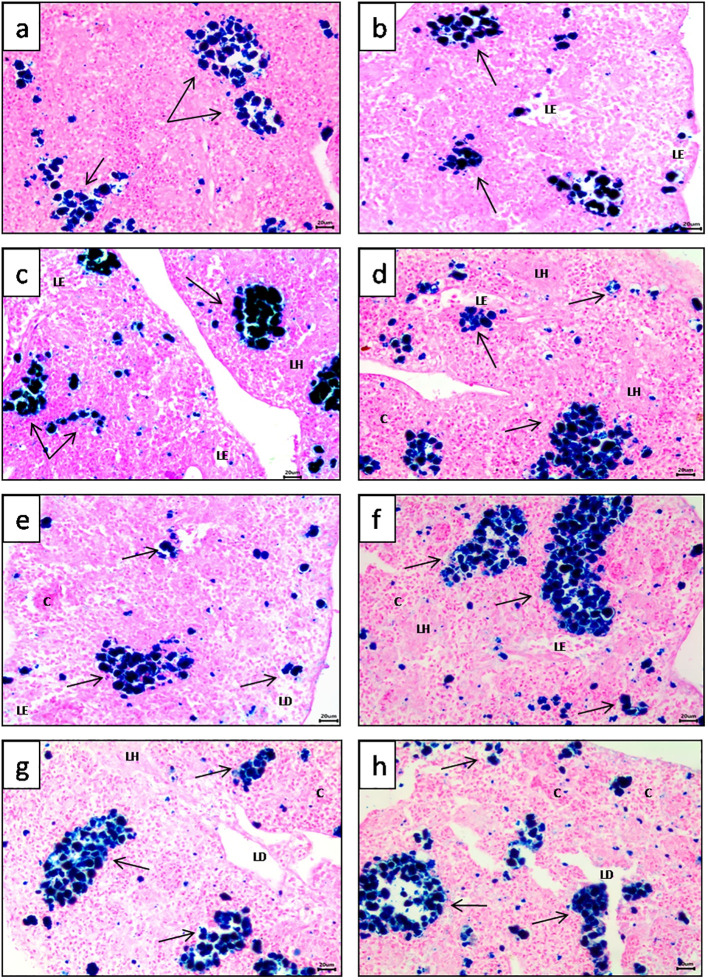
Photomicrographs of Prussian blue–stained spleen sections of grass carp larvae (*Ctenopharyngodon idella*) following 14-day exposure to benzo[a]pyrene (B[a]P) at concentrations of 1, 10, or 100 µmol/L. **(A)** Control group showing absence of iron deposition. **(B, C)** Moderate hemosiderosis (iron deposits, blue color) after treatment with 1 µmol/L. **(D, E)** Moderate degree of iron deposition after treatment with 10 µmol/L. **(F–H)** Severe hemosiderosis after treatment with 100 µmol/L. Arrows indicate the locations of iron staining within the tissues. Additional histopathological changes observed include lymphoid exhaustion of white pulp (LE), hyperplasia of lymphoid follicles (LH), lymphocyte depletion (LD), and congestion of red pulp **(C)**.

## 4 Discussion

The present study demonstrated that exposure to B[a]P at concentrations of 1, 10, or 100 µmol/L induced significant hematological, cytotoxic, and genotoxic alterations in grass carp (*Ctenopharyngodon idella*) larvae. These findings are consistent with, and further reinforce, previous reports on B[a]P and other PAHs toxicity in various fish models.

Regarding hematological parameters, the observed declines in RBC count, Hb concentration, and Ht values across all B[a]P exposure groups align with the findings of others (69), who reported similar findings after rockfish, *Sebastes schlegeli* exposed to dietary B[a]P concentration of 1.5 and 2.0 mg/kg for 30 days. Similar anemia-like symptoms and disruption of hematological parameters were observed after exposed to sublethal concentrations of B[a]P in *Clarias gariepinus* (70, 71), White fish (*Rutilus frissi kutum*) (72), and mice (73).

B[a]P disrupts hematological homeostasis in fish primarily through oxidative stress, genotoxicity, and immunotoxic effects (72, 74, 75). Following exposure, B[a]P is metabolized by cytochrome P450 enzymes, producing reactive intermediates and an excess of ROS that attack cellular macromolecules, including those within hematopoietic tissues such as the spleen and kidney (76–79). Oxidative damage to erythrocyte membranes leads to increased fragility and hemolysis, resulting in reduced RBC counts, Hb concentrations, and Ht levels, indicative of anemia (80–82). Moreover, B[a]P -induced DNA damage in hematopoietic stem cells impairs normal erythropoiesis and leukopoiesis, further contributing to hematological abnormalities such as leukopenia and lymphocyte alterations (83). The suppression of WBC production and function compromises the immune defense, making fish more susceptible to infections (76, 84). Additionally, B[a]P’s interference with iron metabolism and erythrocyte antioxidant defenses exacerbates oxidative stress, accelerating erythrocyte apoptosis and degradation (84, 85). Changes in MCV and MCHC during B[a]P exposure indicate alterations in erythrocyte morphology and function. Overall, B[a]P’s hematotoxicity arises from a complex interplay of oxidative stress, genotoxicity, and possible immune-related stress responses, undermining fish physiological resilience.

Apoptotic responses evaluated through AO/EB staining in our study revealed a significant increase in apoptotic erythrocytes in B[a]P -treated groups, particularly at 100 µmol/L. This is consistent with the findings demonstrating that B[a]P exposure induces DNA damage which leads to apoptosis and heart defects in zebrafish at a concentration of 0.1 μM from 2 to 72 h post fertilization (86), B[a]P specifically induced apoptosis in the head, backbone, and tail region of zebrafish (87). Furthermore, exposed to B[a]P (1, 4, and 8 μg/L) in marine medaka (*Oryzias melastigma*) during early development over a 30-day exposure period indicated a potential connection to apoptosis through oxidative and molecular level investigation pathways (24). B[a]P -induced apoptosis in fish erythrocytes results from oxidative DNA damage, mitochondrial dysfunction, and activation of programmed cell death pathways (88, 89). The metabolic conversion of B[a]P produces ROS that attack mitochondrial membranes, leading to cytochrome c release and activation of caspase-dependent apoptotic cascades (90, 91). Concurrently, B[a]P-DNA adduct formation interferes with critical gene expression and triggers p53-mediated apoptotic signaling (88, 92). This combination of oxidative stress and genotoxic stress prompts phosphatidylserine externalization, chromatin condensation, and nuclear fragmentation, hallmarks of apoptosis observable through fluorescence-based AO/EB staining (88).

The genotoxic potential of B[a]P was clearly reflected in the comet assay results, where DNA strand breaks increased significantly with exposure concentration. These results mirror those of (93), who demonstrated that B[a]P exposure induced DNA damage in *Channa punctatus* following the completion of the 96-hour exposure period to B[a]P. Furthermore, DNA breakage was found in the liver and gill of Caspian White (*Rutilus frissi kutum*) after exposure to 50, 100, and 200 ppb of B[a]P for 21 days (72). The marked increase in tail moment scores with higher B[a]P doses strongly supports the notion that B[a]P metabolites, especially BPDE, cause direct DNA interaction and oxidative lesions. The comet assay directly measures the extent of DNA strand breaks in individual cells, providing sensitive detection of genotoxic effects induced by B[a]P. Excessive ROS generated during B[a]P metabolism causes single- and double-strand breaks, oxidized bases, and DNA cross-linking (72, 93). During electrophoresis under alkaline conditions, fragmented DNA migrates from the nucleus, forming a characteristic ‘comet tail’ whose length and intensity correlate with the degree of DNA damage (63, 92). Elevated tail moments observed in B[a]P -exposed fish erythrocytes reflect severe genomic instability, a precursor to mutagenesis, apoptosis, or carcinogenesis.

In addition to DNA strand breaks, the elevated frequencies of nuclear abnormalities, including micronuclei, polykaryocytes, and abnormal erythrocyte morphologies, are consistent with observations by others (93), who reported micronuclei and nuclear abnormalities in the erythrocytes of *Channa punctatus* following the completion of the 96-hour exposure period to B[a]P. In addition to this cytotoxicity, there was increased frequency of micronucleus after exposure to B[a]P in rainbow trout (*Oncorhynchus mykiss*) at a concentration of 2 mg/kg for 7 days (94). Other studies demonstrate that PAHs induce chromosomal instability and nuclear malformations in fish erythrocytes (72, 95–97), reflecting underlying genotoxic and cytotoxic stress.

The types of nuclear abnormalities observed in our study, including sickle cells and micronuclei, further support the concept that B[a]P exposure disrupts DNA replication and mitotic processes. Exposure to B[a]P leads to distinct morphological alterations in RBCs, resulting from oxidative membrane damage and cytoskeletal disruption. ROS attacks the phospholipid bilayer of erythrocyte membranes, inducing lipid peroxidation and causing structural deformities such as crenation, echinocytosis, and spherocytosis (98). Oxidative injury compromises RBC flexibility and survival, increasing the risk of hemolysis and contributing to anemia (66, 81). Structural changes in RBCs not only impair oxygen transport but also signal broader systemic toxicity affecting fish health and survival (93, 99). The formation of nuclear abnormalities reflects errors in chromosomal segregation and structural chromosome instability, serving as early warning signs of potential carcinogenesis (100). The frequency of such abnormalities increases with B[a]P concentration and exposure duration, highlighting the cumulative genotoxic risk posed by persistent environmental contamination. Thus, nuclear abnormalities provide critical insight into the clastogenic and aneugenic mechanisms of B[a]P toxicity in fish (93, 96, 97). The markedly elevated frequency of erythrocyte abnormalities observed at the highest nominal B[a]P concentration may reflect overlapping cytotoxic and genotoxic effects under exposure conditions. Therefore, some observed abnormalities could be associated with generalized cellular degeneration in addition to specific genotoxic responses. Nevertheless, strict morphological scoring criteria were applied to reduce inclusion of staining or necrotic artifacts.

Our results showed severe hepatic and splenic damage in B[a]P-exposed fish. The liver exhibited diffuse vacuolar and fatty degeneration of hepatocytes, dilation and congestion of blood vessels and sinusoids, necrosis, and apoptosis. Vascular injury included vessel wall degeneration and vascular congestion with coagulated blood elements in the central vein and hepatopancreas. The spleen showed lymphoid depletion, white pulp exhaustion, multifocal lymphoid follicle hyperplasia, and congestion of red pulp and vessels. Additionally, large MMCs aggregates contained extensive hemosiderin deposition in all treated groups. These pathological changes are consistent with previously reported manifestations of PAH toxicity in *Rutilus frisii* (Caspian whitefish) and *Oncorhynchus mykiss* (rainbow trout) exposed to B[a]P and related compounds (72, 101).

Similarly, others (102) have demonstrated that exposure of *Cyprinus carpio* to B[a]P in microcosms caused gill hypertrophy, lamellar necrosis and detachment, and hepatocellular damage characterized by nuclear membrane lysis, lipid droplet accumulation, glycogen granule depletion, and lysosomal proliferation. (72) also reported comparable findings in *Rutilus frisii* exposed to B[a]P, including sinusoidal dilation, necrosis, and epithelial hyperplasia. These results align with those of others (103, 104), who described similar hepatic lesions in sea bream (*Sparus aurata*) exposed to B[a]P.

Hepatocyte damage observed in the present study is likely associated with inhibition of protein synthesis, energy depletion, and disturbance of hepatic energy metabolism (105, 106). Vacuolar and degeneration suggest impaired lipid metabolism and mitochondrial dysfunction, as damaged mitochondria fail to provide sufficient energy for lipid breakdown. Hepatic congestion and sinusoidal dilation indicate circulatory disturbances due to vascular endothelial damage, consistent with observations in *Oreochromis niloticus* and *Clarias gariepinus* exposed to petroleum hydrocarbons and other xenobiotics (107). Such vascular changes impede hepatic blood flow, reduce oxygen diffusion, and exacerbate hypoxia, leading to hepatocellular necrosis (108).

The vascular changes in splenic blood vessels, particularly at higher doses, suggest endothelial injury, increased vascular permeability, and impaired blood circulation, which exacerbate inflammation and tissue stress (109, 110). The increased presence of MMCs aggregates and hemosiderin deposition observed across all treated groups, especially in fish exposed to high concentrations, indicates an adaptive response to enhanced phagocytic activity and erythrocyte breakdown (72, 111, 112). Hemosiderin accumulation reflects iron storage resulting from hemolysis and hemoglobin degradation (113). This pigment often occurs alongside lipofuscin granules, a product of oxidative polymerization of polyunsaturated fatty acids (114). Increased iron content in MMCs is considered a protective mechanism against oxidative stress (115). MMCs are widely recognized as bioindicators of aquatic pollution (116) and as sensitive biomarkers of immune activation under PAH contamination (117, 118). Increases in MMC number and size following toxicant exposure have also been documented after exposure to toxins (72, 75). The observed splenic structural damage, including lymphoid depletion and vascular congestion, implies impaired immune competence and heightened vulnerability to infections.

In aquaculture, even low-level B[a]P exposure in larvae can impair growth, weaken immunity, and reduce productivity due to organ dysfunction (119, 120). Early physiological compromise increases mortality, lowers feed efficiency, and raises economic costs (121). Additionally, as grass carp contribute to aquatic weed control and ecosystem balance, sublethal toxicity in this species may have cascading effects on freshwater ecosystem dynamics (48, 49, 52). Therefore, monitoring and regulating B[a]P in aquatic environments is essential to protect fish health, maintain ecological balance, and support sustainable aquaculture. A limitation of the present study is that exposure concentrations were based on nominal rather than analytically confirmed waterborne B[a]P concentrations. Due to the hydrophobic nature of B[a]P, adsorption to tank surfaces and organic material may have reduced dissolved concentrations during exposure. Therefore, the toxicological responses observed should be interpreted as effects associated with nominal exposure conditions rather than precise dissolved concentrations. Future studies incorporating chemical analysis of exposure media are recommended to better characterize actual bioavailable concentrations. Although the present study demonstrated pronounced toxicological effects of B[a]P in grass carp larvae, the exposure concentrations used were substantially higher than most dissolved environmental concentrations reported in aquatic ecosystems. Therefore, the findings should primarily be interpreted within the context of toxicology and hazard identification. Nevertheless, localized contamination hotspots, sediment-associated PAH accumulation, and trophic transfer may increase organismal exposure in heavily polluted environments. Future studies employing environmentally realistic chronic exposure concentrations would further improve ecological risk assessment.

## 5 Conclusion

This study demonstrates that exposure of grass carp larvae (*Ctenopharyngodon idella*) to benzo[a]pyrene (B[a]P) induces pronounced toxic effects across multiple biological levels, with clear implications for immune-associated biomarkers. B[a]P exposure resulted in significant hematological disturbances, including reductions in red and white blood cell counts and alterations in leukocyte profiles, indicating impaired immune-related physiological status. These changes were accompanied by increased erythrocyte apoptosis and marked genotoxic damage, reflecting cellular stress and compromised biological integrity. Histopathological alterations in the liver and spleen further revealed severe tissue injury, including hepatocellular degeneration, vascular disruption, lymphoid depletion, and pronounced melanomacrophage center (MMC) activation with hemosiderin accumulation. These findings highlight disruption of key immune-related organs and biomarkers, suggesting impaired innate immune defense and increased vulnerability to environmental stressors. Overall, B[a]P acts as a potent immunotoxicant that disrupts immune-related homeostasis, induces cytogenotoxic damage, and compromises tissue integrity in fish larvae under nominal exposure conditions. Under controlled experimental exposure conditions, these results provide important insight into how B[a]P can influence immune-associated responses, cytogenotoxicity, and tissue integrity in fish larvae.

## Data Availability

The raw data supporting the conclusions of this article will be made available by the authors, without undue reservation.
